# Artificial Intelligence and Digital Workflow in Craniofacial Bone Tissue Engineering: From Cone-Beam Computed Tomography (CBCT) to Personalized Bioceramic Implants

**DOI:** 10.3390/dj14090558

**Published:** 2026-09-02

**Authors:** Amirhossein Bahador, Seyed Ali Mostafavi Moghaddam, Hamid Mojtahedi, Lotfollah Kamali Hakim, Hamid Tebyaniyan

**Affiliations:** 1 UCLA School of Dentistry, University of California, Los Angeles, CA 90095, USA; 2 School of Dentistry, Tehran University of Medical Sciences, Tehran 1416634793, Iran; 3 School of Dentistry, Western University of Health Sciences, Pomona, CA 91766, USA; 4 Oral and Maxillofacial Surgery Department, Craniomaxillofacial Research Center, Tehran University of Medical Sciences, Tehran 1416634793, Iran; 5 Department of Oral and Maxillofacial Surgery, School of Dentistry, AJA University of Medical Sciences, Tehran 1411718541, Iran; 6 Science and Research Department, Islimic Azade University, Tehran 1584715414, Iran

**Keywords:** artificial intelligence, digital workflow, CBCT, bone tissue engineering, craniofacial reconstruction

## Abstract

**Background:** Reconstruction of craniofacial bone defects caused by tumor removal, infection, congenital anomalies, or trauma remains a major challenge. Recent advances in artificial intelligence (AI), digital imaging, and additive manufacturing have enabled personalized treatment strategies. This review discusses AI-driven workflows from cone-beam computed tomography (CBCT) image acquisition to implant fabrication using patient-specific bioceramics. **Methods:** Several electronic databases were searched, including Scopus, PubMed, ScienceDirect, and Web of Science. A peer-reviewed article published between 2015 and 2026 was considered for inclusion. This review provides an overview of AI applications in craniofacial tissue engineering, including CBCT image segmentation, three-dimensional reconstruction, virtual surgical planning, computer-aided design/computer-aided manufacturing (CAD/CAM), topology optimization, finite element analysis, and three-dimensional bioceramic scaffold printing. **Results:** AI improves accuracy, efficiency, and reproducibility of craniofacial reconstructions when used in conjunction with AI-assisted workflows. Through automated image segmentation and anatomical modeling, operator dependence can be reduced, and predictive algorithms can optimize biomechanical properties. CAD/CAM and 3D printing are used to manufacture bioceramic implants with controlled porosity, mechanical integrity, and enhanced osteoconductive properties. AI-driven predictive models have demonstrated potential for supporting material selection, manufacturing quality control, and treatment planning; however, their ability to reliably predict long-term postoperative outcomes requires further validation through prospective clinical studies. Data standardization, algorithm transparency, regulatory approval, clinical trial validation, and ethical considerations remain challenges. **Conclusions:** AI, CBCT imaging, computational modeling, and advanced bioceramic manufacturing are being combined to create an end-to-end digital workflow for personalized reconstruction. Clinical studies must be conducted prospectively to maximize the therapeutic potential of these technologies.

## 1. Introduction

Several clinical challenges arise from craniofacial bone defects caused by trauma, congenital conditions, infections, tumor removals, and degenerative diseases. Reconstruction must not only restore skeletal continuity but also facial aesthetics, mastication, speech, and neurosensory function. However, autogenous bone grafting is limited by donor-tissue availability, donor-site morbidity, extended operating times, unpredictable graft resorption, and increased healthcare costs, despite its excellent osteogenic, osteoinductive, and osteoconductive properties. Incorporating biomaterials, stem cells, growth factors, and advanced manufacturing techniques, craniofacial BTE has become more attractive [1,2]. Digital medicine has significantly impacted craniofacial reconstruction. Instead of conventional imaging and manual implant fabrication, clinicians use fully digital workflows to develop patient-specific regenerative solutions. This category includes imaging, CAD, simulation, and additive manufacturing. These digital technologies have improved surgical planning accuracy, implant adaptation, and workflow efficiency; however, the magnitude of their clinical benefit depends on further validation through well-designed clinical studies [3,4].

CBCT has become an important imaging modality in craniofacial digital workflows because it provides three-dimensional visualization of mineralized tissues with high spatial resolution and, in many dentomaxillofacial applications, lower radiation exposure compared with conventional multidetector CT. However, radiation dose depends heavily on acquisition parameters, including field of view (FOV), voxel size, exposure settings, scanning protocol, and the specific CBCT system. Therefore, optimizing the balance between image quality and radiation protection remains essential, particularly when CBCT datasets are used for AI-based segmentation and patient-specific implant design. Although CBCT provides valuable information regarding bone morphology, cortical thickness, trabecular architecture, and defect geometry, quantitative assessment of bone mineral density remains challenging because scanner characteristics, acquisition parameters, reconstruction algorithms, and artifact conditions influence CBCT gray values. Unlike conventional CT, CBCT values cannot be directly interpreted as standardized Hounsfield units. Therefore, CBCT-based density evaluation should be considered semi-quantitative unless calibration methods and validated protocols are applied [5,6]. AI, particularly machine learning, is also transforming digital workflow. AI algorithms can automatically segment CBCT datasets, identify pathological lesions, reconstruct 3D anatomical models, and reduce manual processing. Deep neural networks can enhance clinical workflow by segmenting teeth, detecting mandibular canals, identifying craniofacial landmarks, quantifying bone defects, and enhancing images. Beyond image analysis, AI is now used for predictive modeling, surgical decision support, biomaterial selection, scaffold optimization, and postoperative outcomes [5,7]. Integrating AI with CAD/CAM has revolutionized implant development. Digital anatomical models can be acquired using a CBCT scanner, segmented and optimized using generative design algorithms, finite element analysis (FEA), and topology optimization. This computational tool not only improves structural performance but also optimizes implant geometry, stress distribution, porosity, and mechanical stability [8,9].

Advances in additive manufacturing, specifically 3D printing, have further enhanced craniofacial tissue engineering. Three-dimensional printing can be used to design scaffolds that mimic the macro- and microarchitecture of a patient’s bones as closely as possible. Bioceramic materials have gained considerable attention due to their high biocompatibility, osteoconductivity, bioactivity, and ability to promote bone regeneration. AI-assisted scaffolds and optimized printing parameters further improve the mechanical, degradation, and biological properties of personalized regenerative implants [10,11]. Despite these remarkable advancements, several challenges have hindered the widespread adoption of these technologies. Datasets with diverse annotations are necessary to make AI models robust and generalizable. Regulatory approval, cybersecurity, data privacy, ethical governance, manufacturing standardization, and long-term clinical validation remain significant barriers. To integrate AI-driven regenerative technologies into routine craniofacial surgery, standardized digital workflows and multicenter validation studies are essential [8,12].

Despite the growing number of reviews on bioceramics and craniofacial tissue engineering, most have focused on biomaterial development, scaffold fabrication, or individual aspects of regenerative medicine. Our previous review similarly emphasized personalized bioceramic scaffolds, including their materials, manufacturing strategies, and clinical translation. In contrast, the present review takes a broader perspective by bringing together the complete AI-driven digital workflow for craniofacial bone tissue engineering. It discusses how CBCT, AI, virtual surgical planning, CAD/CAM, finite element analysis, additive manufacturing, and patient-specific bioceramic implants are integrated into a continuous workflow from diagnosis to postoperative evaluation. In addition, this review summarizes the latest advances reported through 2026, including digital twins, intelligent manufacturing, and AI-assisted decision-making, highlighting how these technologies are shaping the next generation of personalized craniofacial reconstruction. By focusing on the integration of digital technologies rather than biomaterials alone, this review provides a complementary perspective with minimal overlap with our previous publication. This review provides an overview of the AI-enabled digital workflow for craniofacial bone tissue engineering, beginning with CBCT image acquisition and continuing through AI-assisted image analysis, virtual surgical planning, computational implant design, finite element optimization, additive manufacturing, and the development of personalized bioceramic implants. It also discusses current challenges, regulatory considerations, and future directions for translating these technologies into routine clinical practice.

## 2. Methods

This review provides a comprehensive overview of recent advances in AI-driven digital workflows for craniofacial bone tissue engineering. The review focused on key components of the digital workflow, including CBCT, AI-based image segmentation, virtual surgical planning, CAD/CAM, finite element analysis (FEA), additive manufacturing, and the development of personalized bioceramic implants.

This review used four major electronic databases: Web of Science, ScienceDirect, PubMed/MEDLINE, and Scopus. Articles published between 2015 and 2026 were considered for inclusion. The search strategy combined Medical Subject Headings with free-text keywords using Boolean operators to maximize the retrieval of relevant studies. Representative search terms included: (“artificial intelligence” OR AI OR “machine learning” OR “deep learning”) AND (“cone beam computed tomography” OR CBCT) AND (“craniofacial” OR maxillofacial OR mandibular OR cranial) AND (“bone tissue engineering” OR bone regeneration OR scaffold OR biomaterial OR bioceramic) AND (“digital workflow” OR CAD/CAM OR “virtual surgical planning” OR “finite element analysis” OR “3D printing” OR “additive manufacturing” OR “patient-specific implant”). In addition, we manually screened the reference lists of eligible articles and relevant review papers to identify any additional studies that may have been missed during the electronic search.

Studies were considered eligible if they were published in peer-reviewed journals, written in English, and published between 2015 and 2026. Eligible publications investigated one or more aspects of AI-assisted craniofacial bone tissue engineering, including CBCT-based image analysis, digital workflows, CAD/CAM, virtual surgical planning, computational modeling, finite element analysis, additive manufacturing, or personalized bioceramic implants. Original research articles, clinical and preclinical studies, technical reports, and high-quality review articles providing relevant background information were included.

Studies were excluded if they were editorials, letters, patents, duplicate publications, or articles lacking sufficient methodological or technical detail. Publications not written in English were also excluded.

After removing duplicate records, titles and abstracts were independently screened for relevance. The full texts of potentially eligible studies were then assessed against the predefined inclusion and exclusion criteria. Only studies directly related to AI-assisted craniofacial reconstruction, digital workflows, and patient-specific bioceramic implant design were included in the final review.

The initial search identified approximately 308 publications. After the screening and eligibility assessment, 119 articles met the inclusion criteria and were included in the final analysis. PRISMA was not followed. Data extracted from the included studies included publication year, study design, AI methodology, imaging modality, computational workflow, biomaterials, additive manufacturing techniques, and the principal findings related to craniofacial bone regeneration. The findings were synthesized narratively and organized into thematic sections that reflect the complete AI-enabled digital workflow, from CBCT image acquisition and automated image analysis to virtual surgical planning, patient-specific implant design, additive manufacturing, and clinical translation. Given the broad scope of this narrative review and the heterogeneity of the included study designs, a formal quality assessment or risk-of-bias analysis was not performed. Instead, emphasis was placed on recent, peer-reviewed publications from reputable scientific journals that provided robust and clinically relevant evidence on AI-assisted digital workflows and craniofacial bone tissue engineering.

## 3. Craniofacial Bone Tissue Engineering

The craniofacial tissue complex plays an important role in chewing, smiling, and speaking. The complex has an aesthetic appeal that may affect psychological well-being as well as facial function. Damage to craniofacial tissues can affect a person’s physical and social health, so accurate reconstruction is vital to restoring their functionality and aesthetic appeal [13,14,15]. Biomaterial scaffolds, MSCs, and growth factors work synergistically during craniofacial BTE. By combining the two models, critical-size cranial defects can be repaired to promote new bone formation. Various alternatives have also shown promise, including alveolar ridges and maxillary sinus floors. Numerous clinical studies have used allograft scaffolds derived from calvarial bone and mandibular condyles. Critical-size bone defects require several steps to repair. As part of the implanted scaffold, bone cells should be delivered and recruited to the defect site; growth factors should be incorporated into biomaterial scaffolds; vascularization, bone tissue formation, in vivo oxygen and nutrient exchange, and scaffold degradation should be balanced and supported. Bone regeneration in the craniofacial region appears to require these requirements [14,16,17]. A prominent position makes the craniofacial region susceptible to trauma that can lead to defects that require surgery and reconstruction. Several graft types are used to repair and regenerate craniofacial defects, including autografts, allografts, xenografts, and alloplasts. In addition to being immunologically inert, autografts possess osteogenic and osteoconductive potential, making them an excellent option for craniofacial reconstruction. Autogenous bone grafts, however, have several disadvantages, including donor site morbidity [18,19].

Craniofacial bone regeneration can be achieved by using autogenous bone grafts from iliac crests and ribs. Craniofacial bones and other long bones have germ layers, which are the origins of the long bones. Bone graft availability is limited, and donor-site morbidity is also a concern. The graft may resorb if it is not vascularized adequately. Revascularization of the grafted bone occurs after implantation, preserving the periosteal layer and the surrounding environment. In contrast to long bones, craniofacial bones have a more complex 3D structure, making reshaping them more challenging. A practical way to treat craniofacial bone defects is to synchronize active constituents, cells, and growth inducers, as this can overcome limitations in availability and the complex 3D structures. An experimental stem cell treatment for craniomaxillofacial defects proved successful in a clinical trial. Studies have found that a similar approach is effective in preventing osteonecrosis of the femoral head and distal tibial fractures [13,20].

Because craniofacial bone tissue engineering has several limitations, 3D-bioprinted craniofacial bone implants have gained significant popularity. In 3D bioprinting, stem cells, scaffold materials, and growth factors are used to fabricate tissues. Bioprinting and 3D printing are sometimes confused. It is often advisable to use inert scaffolds when printing live cells or to print dense cells without a scaffold. Several reports confirm that 3D bioprinting can revolutionize craniofacial bone tissue engineering. Patients with congenital or trauma-related craniofacial defects can benefit from 3D-bioprinted craniofacial bones. By printing implants matched to the patient’s anatomy, CAD programs can ensure excellent implant–bone contact. There is still a long way to go in bioprinting development. Identifying suitable biocompatible materials to support the growth and function of craniofacial BTE using 3D bioprinting technology remains challenging. Additionally, the crosslinking patterns of the materials must be adequate to ensure accurate deposition, bioactivity, and lasting strength [13,14,21].

In recent years, BTE has advanced to the point where porous materials are being developed, designed, and manufactured to restore the natural function and condition of damaged parts. Bioceramics and their composites have proven to be important materials for developing bone scaffolds. Recent advances in 3D bioprinting techniques have enabled the fabrication of complex scaffolds, including those for craniofacial bones. Although bioceramics research has advanced, its limitations have prevented its full potential from being realized. The field of ceramic chemistry is undergoing several exciting developments. Using it, researchers have formed tissue-engineering composites with glass. Glass composites support bone development through improved tissue interactions. A scaffold that stimulates bone regeneration is ideal. Cells should be able to move through it if its pore size is small enough. This glass is bioactive, and nanoporosity allows it to control degradation rates [13,14,22].

For most 3D printing techniques, ceramics present a serious challenge because their melting points and optical and mechanical properties make them unsuitable for most current techniques. Ceramics are created by polymerizing ceramic powders and hardening them under light. A typical printed material will contain as much as 50 percent ceramic powder. Because of the bond between the ceramic powder and the plastic, the printed material lacks the desirable ceramic properties of strength and hardness. After the plastic is analyzed, oxidized, and volatilized, high-purity ceramics are produced by heating printed objects to high temperatures, causing them to shrink as the plastic decomposes. As biomaterials for orthotics are increasingly used, they have elicited specific biological responses in several industrial applications. A bioceramic material has excellent physical and chemical properties, as well as excellent biocompatibility. Their low cost and ease of manufacture make them popular in regenerative medicine [23,24].

## 4. AI in Craniofacial Bone Medicine

AI can help design and fabricate bone scaffolds more efficiently. AI-driven generative design and predictive modeling improve the mechanical properties, biocompatibility, and porosity of bone scaffolds. Combining additive manufacturing with AI further supports defect detection, optimization, and strategic material selection [25,26]. Biomaterials and closed-loop manufacturing systems are making these scaffolds more effective and sustainable. However, the primary challenges and technological advances in scaffold design are as follows. In resource-constrained areas, AI and additive manufacturing can also reduce production costs [25,27]. Computers can emulate human cognitive processes, enabling them to think, reason, and perceive as humans do. Unlike humans, computers can perform complex calculations accurately and efficiently. AI includes several subfields, including machine learning and natural language processing. In machine learning, data analysis continually improves through experience using specialized algorithms. A neural network is the basis of deep learning [28]. With AI, relevant information can be extracted from data, and clinical decisions can be supported, resulting in greater efficiency and convenience for clinicians. Applications include biomaterials used in artificial bone substitutes, drug discovery, and radiology imaging. Biomaterial discovery and reuse can be accomplished by mining biomedical databases and clinical research experiments that are neither visible nor reproducible. The number of publications related to AI and bone regeneration has increased. According to a novelty search, a relatively small number of relevant reviews were found across various databases on AI screening for bone regeneration materials, as most studies did not focus on bone regeneration [29,30].

AI-based computational approaches have emerged as valuable tools for predicting biomaterial properties and guiding material discovery. However, these models currently complement rather than replace experimental validation, as in vitro and in vivo studies remain essential for confirming biological responses, safety, degradation behavior, and clinical applicability. By applying AI, many high-cost experiments have been replaced, opening a new pathway for screening bone regeneration materials. To evaluate and screen materials promptly, AI can simulate their behavior through computer modeling. Therefore, finite-element models and in vitro experiments were used to determine the mechanical properties of bone biomaterials. AI is now working to discover and synthesize new materials with excellent properties. An ideal material should be biocompatible, mechanically strong, antibacterial, and noncytotoxic. The inside of human bone tissue should be porous, allowing human tissues to grow and enter. Materials can release cytokines and growth factors to promote bone tissue growth. By interacting with bone, implants can promote bone regeneration by inducing vascularization and nerve regeneration in surrounding bone tissue. During the first year or two after implantation, they can rapidly degrade, and new bone tissue gradually replaces them. Based on AI evaluation of material properties, this section provides an overview of AI’s outstanding contributions to bone regeneration [29,31].

In addition to fractures, age-related diseases, tumors, and autoimmune diseases, bone disorders can be treated with drugs and transplants. However, these methods are often ineffective, associated with numerous side effects, and not cost-effective. The use of tissue engineering to regenerate bone defects has increased in recent years. BTE promotes bone tissue regeneration by combining biological and engineering properties. A successful tissue engineering strategy requires three essential ingredients: scaffolds, cells, and growth factors that act as osteoconductive, osteogenic, and osteoinductive factors [32,33]. Optimizing scaffold properties can dramatically enhance osteogenesis and osteoinduction. Computational models, however, can be useful tools for predicting how well scaffolds regenerate bone and for designing scaffolds that are well organized for bone regeneration. A scaffold can be optimally designed by predicting cellular processes in the body’s environment. Computational modeling studies have shown that it can predict bone formation, although it is currently limited to a small number of unit cells within scaffolds. Scaling across a range of spatial dimensions is prohibitively expensive due to the computational costs involved [32,34].

Multilevel finite element (FE) methods can analyze scaffolds at both macro- and microscales, but implementing them for tasks such as design optimization is difficult. For these scenarios, FE computations must be iterated and/or repeated, incurring prohibitive computational costs. Studying the micro- and macro-interactions of cells in BTE can facilitate preclinical-to-clinical translation. Therefore, using AI to predict these interactions is extremely practical for tissue engineering [32,35]. Javaid et al. (2023) [36] examined two main contributions to the study. They identified initial biomaterials for bone scaffolds using machine learning models. Second, they used PROMETHEE to rank the best biomedical scaffold materials based on preference rankings. Cortical bone is used as a reference for determining comprehensive strength, tensile strength, and compressive strength using Young’s modulus. Because ANN outperformed other machine learning models, it identified brushite and titanium alloy as the most suitable biomaterials for bone tissue engineering [36]. Table 1 summarizes common studies investigating AI applications and digital workflows in craniofacial bone tissue engineering.

Bermejillo Barrera and colleagues (2021) validated the 3D CNN for predicting the mechanical properties of innovative scaffolds. AI- or ML-aided design strategies were considered novel approaches for tissue engineering scaffolds and mechanical metamaterials. A 3D convolutional neural network trained on digital tomographies of CAD models was proposed for tissue-engineering scaffolds. Beyond tissue engineering, this methodology can be applied to regenerative medicine and mechanical metamaterials to predict the mechanical properties of complex scaffolds [37]. Robles-Bykbaev et al. (2019) [38] proposed an imaging-based method incorporating machine learning and statistical learning to model and estimate osteoblast and collagen growth. They developed models to examine relationships among osteocyte growth and differentiation from stem cells and collagen degradation. As a scaffold for bone regeneration, collagen biodegradation was characterized and parameterized using imaging, machine learning, and statistical tools. Biological activity-induced mass loss was estimated with a parametric logistic mixture model. Additionally, the proposed methodology can estimate the degree of scaffold degradation using optical microscopy data [38].

**Table 1:** Representative studies investigating AI applications and digital workflows in craniofacial bone tissue engineering.

Study/Year/Ref	Aim			Outcomes
UNetSPSR study/2026/[39]	Develop and validate a super-resolution model based on a generative adversarial network (UNetSPSR) that improves visualization of fine anatomical structures while minimizing CBCT noise.	**Study design**	Original experimental AI imaging study	With UNetSPSR, image quality was best, with higher PSNR and LPIPS scores than those of existing methods. Using this model, fine anatomical structures improved, trabecular thickness bias dropped from 61% to 11%, microarchitectural measurements improved significantly, and root canal segmentations became more accurate. UNetSPSR improves CBCT image resolution while preserving anatomical structures, showing promising clinical potential.
		**Sample size/Dataset**	CBCT datasets from 1 human skull, 4 cadaveric heads, and 6 sheep heads; independent clinical/public datasets for evaluation	
		**AI model/Digital workflow**	GAN-based super-resolution model (UNetSPSR) for CBCT enhancement	
		**Validation method**	Comparison with existing reconstruction methods; anatomical segmentation assessment	
		**Performance metrics**	PSNR, LPIPS; reduction in trabecular thickness bias; improved root canal segmentation	
		**Evidence level**	Low–moderate (preclinical AI validation)	
		**Principal limitations**	Limited biological datasets; need for broader multicenter validation; unclear clinical impact	
AI applications in CBCT dentistry review/2023/[40]	This review presents recent applications of AI in CBCT to improve diagnosis and treatment planning in dentistry.	**Study design**	Narrative/systematic review	Automated detection, classification, and segmentation of oral and craniofacial structures make CBCT imaging more accurate, efficient, and diagnostically valuable. CBCT imaging with AI remains challenging and limited despite its promising clinical applications.
		**Sample size/Dataset**	Multiple published AI-CBCT studies.	
		**AI model/Digital workflow**	Machine learning, deep learning, segmentation, detection, super-resolution techniques	
		**Validation method**	Literature-based evaluation	
		**Performance metrics**	Reported diagnostic and segmentation performance from included studies	
		**Evidence level**	Moderate (evidence synthesis)	
		**Principal limitations**	Heterogeneity of AI models and datasets; lack of standardized validation	
CNN-based craniomaxillofacial segmentation study/2024/[41]	An automated approach using convolutional neural networks (CNNs) for integrated segmentation of craniomaxillofacial structures from CBCT scans will be compared with a semi-automated approach to assess accuracy, time efficiency, and consistency.	**Study design**	Original retrospective imaging study	Based on CNN, we achieved better segmentation accuracy. Processing time was reduced substantially, and greater consistency was demonstrated. In most cases, refinements corrected undersegmentation of the thickening sinus mucosa and thin bones. For generating virtual patients from CBCT, the automated approach proved accurate, rapid, and reliable.
		**Sample size/Dataset**	30 CBCT scans	
		**AI model/Digital workflow**	CNN-based automated segmentation of craniofacial structures	
		**Validation method**	Comparison with reference models and expert refinement	
		**Performance metrics**	Segmentation accuracy, processing time, consistency	
		**Evidence level**	Low–moderate	
		**Principal limitations**	Small sample size; limited external validation	
AI segmentation with metallic artifacts study/2026/[42]	Analyze the feasibility and accuracy of segmenting CBCT images using AI to generate three-dimensional virtual patients when metallic artifacts are present.	**Study design**	Original experimental imaging validation	Using AI-based segmentation, it created 3D virtual patient models with RMS values between 0.17 and 0.30 mm and near-zero MSD values. Segmentation accuracy was high in the mandible and mandibular teeth, whereas metallic artifacts in zirconia crowns led to higher deviations. However, expert clinical supervision remains necessary for AI-based CBCT segmentation.
		**Sample size/Dataset**	Four different crown-placement conditions, two CBCT systems. Phantom CBCT datasets with zirconia crowns	
		**AI model/Digital workflow**	AI-based segmentation of teeth, mandible, and restorations	
		**Validation method**	Comparison with intraoral scans and digital crown models	
		**Performance metrics**	RMS deviation, median surface deviation (MSD)	
		**Evidence level**	Low	
		**Principal limitations**	Phantom model rather than clinical population; metallic artifact challenges remain.	
CBCT/CAD/CNC bone graft workflow review/2025/[43]	A systematic literature review of CBCT, CAD, and CNC-assisted bone-grafting workflows in oral and maxillofacial surgery is presented.	**Study design**	Systematic review	The reviewed studies demonstrated good results in graft adaptation, volumetric stability, and implant survival with CAD/CAM-customized grafts made from synthetic, allogeneic, xenogeneic, and autogenous materials. It remains unclear how a customized biological bone block is manufactured and implanted after CBCT acquisition; however, no study has reported the entire clinical workflow. Unvalidated digital-to-biological protocols remain the principal barrier to future clinical translation.
		**Sample size/Dataset**	16 included studies plus historical studies	
		**AI model/Digital workflow**	Digital planning, CAD/CAM, CNC-assisted graft fabrication	
		**Validation method**	Clinical and preclinical evidence synthesis	
		**Performance metrics**	Graft adaptation, volumetric stability, implant survival outcomes	
		**Evidence level**	Moderate	
		**Principal limitations**	Lack of a complete validated digital-to-biological workflow	
Digital workflow for horizontal ridge augmentation/2023/[44]	Developing and evaluating a surgically accurate and clinically efficient digital workflow for onlay bone grafting in horizontal alveolar ridge reconstruction.	**Study design**	Prospective pilot clinical study	No complications were associated with any of the bone grafts or implants. According to our results, the average RMSE was 0.41 ± 0.15 mm, indicating high surgical accuracy. According to surgeons, the workflow demonstrated accuracy and clinical feasibility since it was intuitive, easy to understand, and helpful.
		**Sample size/Dataset**	8 patients with horizontal alveolar defects	
		**AI model/Digital workflow**	CBCT-based virtual planning, surgical guide, CAD workflow	
		**Validation method**	Postoperative accuracy and clinical assessment	
		**Performance metrics**	RMSE and implant outcomes	
		**Evidence level**	Moderate	
		**Principal limitations**	Small clinical cohort; short-term evaluation	
Hollow-strut diopside scaffold study/2023/[45]	A hollow-strut diopside bioceramic scaffold with controllable channel geometries is being developed using extrusion-based additive manufacturing.	**Study design**	Experimental in vitro/material study	It was found that hollow-strut scaffolds exhibited sufficient porosity and mechanical strength to repair osteoporotic bone. Square hollow-strut scaffolds demonstrated higher compressive strength despite lower density because of reduced stress concentration. Because of the hollow channels, cells attached and proliferated much better, indicating improved osseointegration and greater bone regeneration potential.
		**Sample size/Dataset**	3D-printed scaffold models	
		**AI model/Digital workflow**	Extrusion-based additive manufacturing with computational optimization	
		**Validation method**	Mechanical testing, finite element analysis, cell evaluation	
		**Performance metrics**	Porosity, compressive strength, cell attachment/proliferation	
		**Evidence level**	Low	
		**Principal limitations**	In vitro study; limited translation to clinical application	
Customized bioengineered scaffold review/2025/[46]	This review presents the development and clinical translation of customized bioengineered implant scaffolds for tissue regeneration, focusing on craniofacial reconstruction, biomaterial choice, manufacturing strategy, and regulatory considerations.	**Study design**	Review article	In addition to improving patient-specific tissue regeneration and surgical complexity, customized bioengineered scaffolds may also enhance clinical outcomes. In addition to identifying key factors for successful clinical translation, the review highlights challenges that must be overcome before routine clinical implementation.
		**Sample size/Dataset**	Published clinical and preclinical studies	
		**AI model/Digital workflow**	AI, 3D printing, biomaterials, GMP workflows	
		**Validation method**	Literature evaluation	
		**Performance metrics**	Reported clinical translation parameters	
		**Evidence level**	Moderate (evidence synthesis)	
		**Principal limitations**	Limited clinical trials and regulatory evidence	
AI-assisted additive manufacturing review/2025/[47]	This review examined recent advances in additively manufactured bone implants, focusing on design strategies, materials, manufacturing processes, and performance evaluation for orthopedic reconstruction.	**Study design**	Review article	Additively manufactured bone implants can improve anatomical matching and functional outcomes. Biomaterials, intelligent manufacturing, and AI -based prediction models have improved implant performance and support the development of future complex bone repair systems.
		**Sample size/Dataset**	Published implant manufacturing studies	
		**AI model/Digital workflow**	AI-based design optimization and intelligent manufacturing	
		**Validation method**	Literature-based assessment	
		**Performance metrics**	Implant performance, design optimization outcomes	
		**Evidence level**	Moderate	
		**Principal limitations**	Limited clinical validation of AI-based manufacturing	
CBCT AI artifact reduction systematic review/2026/[48]	CBCT image quality and artifact reduction should be systematically evaluated using AI-based approaches.	**Study design**	Systematic review	Studies examined metal artifact reduction, scatter correction, image reconstruction, motion artifact reduction, and noise reduction. The most commonly used U-Net-based models generally improved CBCT image quality. Several factors limit conclusions about the clinical applicability of these AI-based approaches, including considerable variability in evaluation methods, limited availability of public datasets and source code, and insufficient diagnostic validation.
		**Sample size/Dataset**	27 studies	
		**AI model/Digital workflow**	U-Net and other AI reconstruction models	
		**Validation method**	QUADAS-II quality assessment	
		**Performance metrics**	Image quality metrics, artifact reduction performance	
		**Evidence level**	Moderate	
		**Principal limitations**	Variable methodologies; limited public datasets and diagnostic validation	
3D-printed bioceramic scaffold review/2024/[49]	The review focuses on the role of 3D-printed bioceramic scaffolds in bone regeneration, immune regulation, and treatment of large bone defects.	**Study design**	Narrative review	3D-printed bioceramic scaffolds provide customizable architectures that promote bone regeneration and modulate immune responses. The scaffold’s optimized material composition and structural features promote bone regeneration and modulate immune responses. Despite the promising results, challenges remain in materials, printing precision, scaffold stability, and clinical translation, underscoring the need for improved biomaterials and advanced fabrication techniques.
		**Sample size/Dataset**	Published biomaterial studies	
		**AI model/Digital workflow**	Extrusion printing, FDM, light curing, bioceramic scaffolds	
		**Validation method**	Literature synthesis	
		**Performance metrics**	Biological performance and scaffold characteristics	
		**Evidence level**	Moderate	
		**Principal limitations**	Mainly preclinical evidence; clinical adoption remains limited	

## 5. CBCT as the Foundation of the Digital Workflow

Digital workflows in craniofacial BTE rely on CBCT because it provides high-resolution 3D visualization of craniofacial hard tissues with lower radiation exposure and cost. By integrating CBCT with AI, CAD/CAM, FEA, and additive manufacturing, patient-specific implants can now be designed, planned, and manufactured more efficiently and effectively. Now that CBCT is no longer merely a diagnostic imaging modality, it serves as the primary source of anatomical data for image segmentation, virtual surgical planning, and 3D printing of customized scaffolds [5,50]. In a typical AI-driven digital workflow, CBCT images are acquired, preprocessed, segmented, 3D-reconstructed, used for virtual surgical planning, subjected to computational optimization, used for implant fabrication, and evaluated postoperatively. Accurate volumetric imaging is essential for precision craniofacial reconstruction. As deep learning advances, CBCT-derived datasets are becoming more accurate, with fewer artifacts, improved resolution, and automated image-processing tasks. Consequently, clinician workload has been reduced, and diagnostic consistency has improved [5,51].

Multiple advantages make CBCT more advantageous for craniofacial applications than conventional multidetector CT. Scanning time, radiation dose, and operational costs are reduced. For osseous structures, the isotropic voxels provide great spatial resolution. CBCT systems make it easier for dentists and maxillofacial surgeons to acquire patient-specific anatomical information. CBCT enables highly accurate replication of complex craniofacial anatomy with custom implants and tissue-engineering scaffolds. CBCT’s inherent limitations can adversely affect image interpretation, including poor contrast between soft tissues, metal artifacts, and gray-value variability. To address these limitations and expand the use of CBCT in regenerative medicine, AI is increasingly used [50,52]. By integrating AI with manual processes, CBCT has become more intelligent and automated. With deep convolutional neural networks (CNNs), U-Net architectures, transformer-based models, and hybrid machine learning algorithms, anatomical structures can now be identified accurately and reproducibly. These algorithms reduce manual intervention and rapidly generate digital anatomical models, enabling implant customization within a short timeframe. As a result, CBCT has evolved from a diagnostic imaging tool to a digital platform for precision craniofacial reconstruction [53,54].

CBCT acquires multi-dimensional images by rotating a cone-shaped beam and a flat-panel detector around the patient. These projections can be used to reconstruct 3D models of craniofacial structures in three orthogonal planes. In CBCT, the entire region is captured in a single rotation, rather than in sequential slices as in conventional CT [5,55]. With CBCT, cortical bone, trabecular architecture, alveolar bone morphology, and skeletal defects in the maxilla and mandible can be precisely visualized for craniofacial bone tissue engineering. Measurements of bone volume, defect geometry, and anatomical relationships improve virtual surgical planning. From diagnosis to production, CBCT data can be integrated with CAD using the DICOM format [52,56]. High-quality images are essential for AI-assisted digital workflows. Choosing the right field of view (FOV), voxel size, and exposure parameters affects segmentation and implant design accuracy. Voxel size represents an important determinant of CBCT image quality and segmentation accuracy. Smaller voxel sizes provide improved spatial resolution and better visualization of fine anatomical structures; however, they may require increased radiation exposure and can introduce higher image noise if acquisition parameters are not optimized. Conversely, larger voxel sizes may reduce spatial resolution but can improve signal-to-noise characteristics and decrease radiation burden. Therefore, voxel selection should be clinically justified according to the diagnostic objective and the requirements of subsequent digital workflows. Therefore, radiation safety and image quality must be balanced. In recent years, AI-based image enhancement techniques have significantly improved CBCT images. Deep learning algorithms can reduce noise, suppress metal artifacts, enhance contrast, and generate super-resolution images without increasing radiation exposure. This makes it possible to segment and model patients more accurately without compromising safety [5,57].

Image artifacts caused by metallic restorations, orthodontic appliances, patient motion, and beam hardening limit CBCT image quality. Metal restorations, orthodontic appliances, patient movement, and beam-hardening effects remain important limitations of CBCT imaging. Recent AI-based approaches, including convolutional neural networks, generative adversarial networks, and deep learning reconstruction models, have shown potential to reduce noise, correct artifacts, improve contrast, and enhance anatomical visualization. However, these approaches remain dependent on training datasets and require further clinical validation before widespread implementation in routine craniofacial workflows. Using deep learning models trained on paired datasets, anatomical information can be automatically restored, and bone boundaries can be better visualized. In generating CT-like images from CBCT data, generative adversarial networks (GANs) and diffusion models have shown promising performance. Such techniques can enhance quantitative image analysis and improve finite element modeling and implant design [5,58]. Image segmentation is important for digitally reconstructing anatomical structures during implant manufacturing. Manual segmentation requires significant time and effort and is subject to interobserver variability. Digital craniofacial workflows have become increasingly dependent on AI-based segmentation. U-Net, 3D U-Net, Mask R-CNN, and transformer-based networks accurately segment the mandible, maxilla, teeth, mandibular canal, maxillary sinus, and cranial bones from CBCT images. Based on meta-analyses, AI achieves excellent accuracy in segmenting oral and maxillofacial structures while reducing processing time [59,60].

To diagnose orthodontic problems, plan orthognathic surgery, plan implants, and engineer craniofacial tissues, craniofacial landmark localization must be automated. Manual landmark identification is often difficult and inconsistent. Anatomical landmarks can be detected within seconds using deep learning models trained on large CBCT datasets. Automating landmark identification improves reproducibility, standardizes cephalometric analysis, provides reference points for virtual surgical planning, and customizes implant positioning. As a result, clinician workload is significantly reduced while digital treatment planning is more precise [61,62]. A digital model of the patient’s craniofacial anatomy is then generated from segmented CBCT datasets. These models enable simulations, digital reconstructions, finite-element analysis, and fabrication of customized implants. Surface meshes are exported as STLs and integrated into CAD software during implant design and additive manufacturing. The AI process extracts surfaces more accurately, corrects segmentation errors, and facilitates multimodal image fusion. Combining CBCT with intraoral, facial, and optical imaging enables the creation of highly accurate digital twins of the skeleton, dental structures, and facial soft tissues. With these comprehensive virtual models, you can reconstruct your own craniofacial structure and develop precise tissue-engineering procedures [63,64,65].

**Figure 1:**
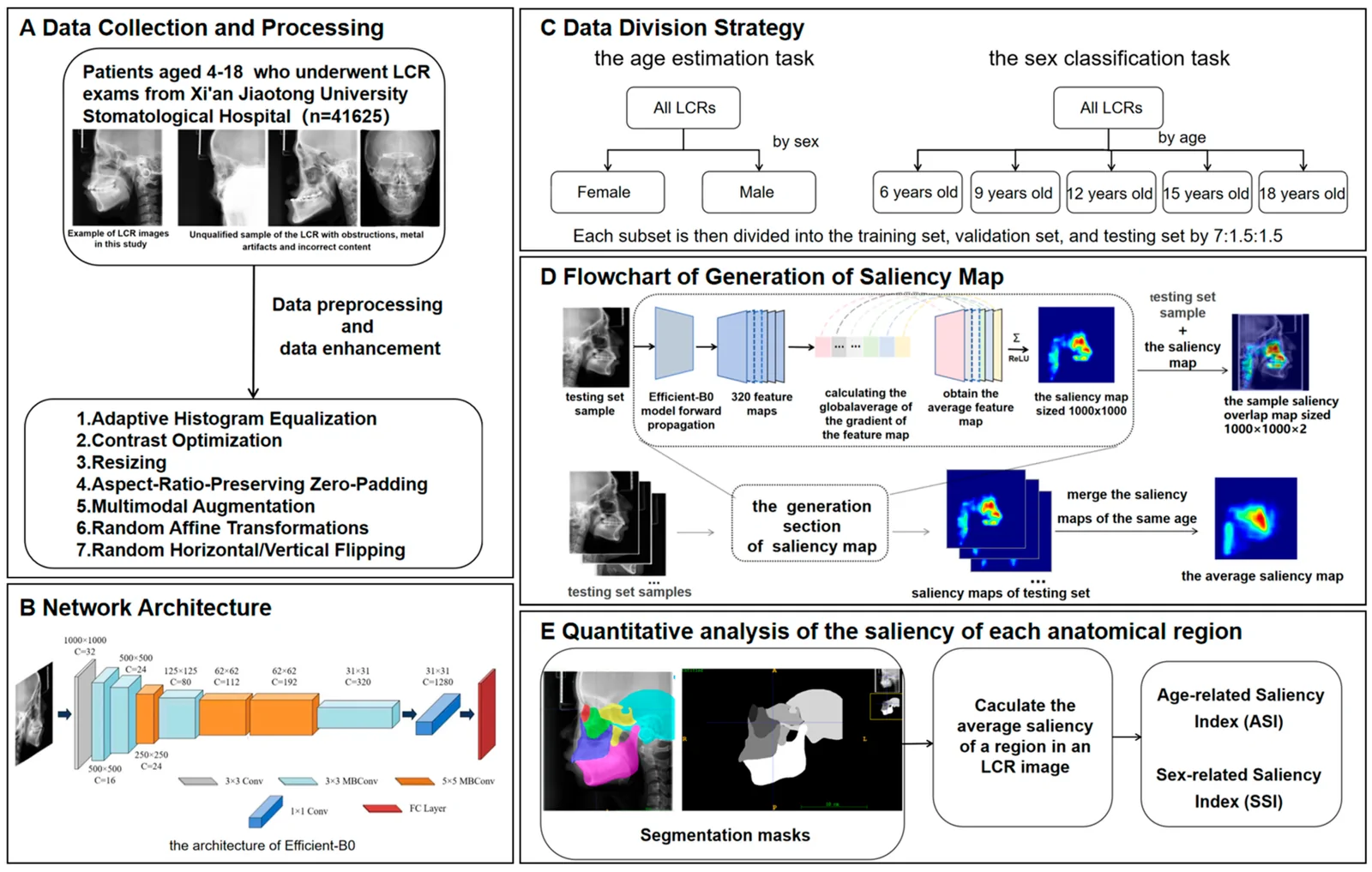
An overview of the study’s methodology. Collected and processed data (**A**). An overview of the network architecture (**B**). Division of data (**C**). An overview of the process of creating a saliency map (**D**). Analyzing the saliency of each anatomical region quantitatively (**E**). Based on the lateral X-ray cephalogram, the saliency map shows the final age-estimation result for each region. In addition to containing age-group feature information, the saliency map is generated using EfficientNet-B0. An overlap map of sample saliency is constructed by concatenating each sample in a test set with its corresponding saliency map of size 1000 × 1000 × 2, where 2 indicates the number of channels. A saliency map corresponding to a particular age is created by merging all saliency maps of the same age. Anatomical regions with more age-related predictive importance are indicated by a more intense red hue on the saliency map [66].

To extract growth and developmental features during learning, Hu et al. (2026) [66] used the EfficientNet-B0 architecture. An AI framework was developed. By using dynamic imaging features spanning continuous age intervals in craniofacial development and discriminating features associated with sexual dimorphism, this model eliminates the need for manual annotation. Grad-CAM visualizes age and gender patterns using saliency maps. The Age-related Saliency Index (ASIA) and the Sex-related Saliency Index were also introduced to evaluate the significance of developmental and dimorphic characteristics in the key craniofacial regions (Figure 1). With age-related saliency maps, multiple bone regions were intuitively visualized during dynamic development, while with the ASI, these bone regions were quantitatively prioritized based on their internal anatomical details. In the evolution of sexual dimorphism, the Sex-related Saliency Index showed initial differences evenly distributed throughout the cranium. Their goal was to develop an end-to-end framework based on lateral cephalometric radiographs. Age- and sex-related average saliency maps were used to visualize and quantify craniofacial growth dynamics. Besides validating established developmental theories, these findings also provide new insights into sex-specific radiographic characteristics of craniofacial bones. Clinicians can use them to assess developmental stages and guide interventions in specific craniofacial regions, as well as to assess sex-specific radiological characteristics [66].

According to Ignac et al. (2026) [67], a 2.5D deep learning model is based on multiplanar CT reconstructions and a ResNet-18 network. The model achieved an AUCROC of 0.822 and an AUCPR of 0.855. According to an independent clinical assessment using Grad-CAM, the visualizations correctly identified fracture-related structures. In light of this study’s findings, radiomics and hybrid deep-radiomics pipelines for pediatric craniofacial trauma can be further developed [67].

## 6. AI-Driven Digital Workflow for Personalized Craniofacial Bone Reconstruction

It is important to distinguish AI from conventional digital technologies used in craniofacial reconstruction. Technologies such as CBCT imaging, CAD/CAM design, virtual surgical planning, FEA, and additive manufacturing constitute the digital infrastructure that enables patient-specific reconstruction but do not inherently represent AI. AI contributes to selected stages of this workflow through machine learning and deep learning algorithms. Specifically, AI is applied for automated CBCT image enhancement, artifact reduction, anatomical segmentation, landmark detection, three-dimensional reconstruction, predictive modeling, generative implant design, biomechanical optimization, and intelligent manufacturing quality control. Therefore, the current AI-driven workflow should be interpreted as integrating AI algorithms with established digital technologies rather than replacing them [68,69,70].

Precision medicine has revolutionized craniofacial bone tissue engineering with digital workflows and AI. AI-enhanced digital workflows integrate AI algorithms with established technologies, including advanced imaging, computer-aided design, biomechanical simulation, additive manufacturing, and postoperative assessment. AI-assisted workflows have shown potential to reduce manual workload and improve reproducibility at several stages of craniofacial reconstruction, particularly image segmentation and computational design. Nevertheless, clinical implementation remains dependent on validation across diverse patient populations and healthcare settings. It reduces surgical planning times, improves surgical predictability, and improves functional outcomes [71,72]. CBCT is used to obtain anatomical data, but conventional computed tomography, magnetic resonance imaging, and intraoral scanning (IOS) may also be used depending on the clinical indication. CBCT has become the imaging modality of choice for craniofacial reconstruction due to its high spatial resolution and low radiation dose. Image quality is improved by removing artifacts, enhancing contrast, and normalizing voxels in DICOM datasets. Convolutional neural networks (CNNs) can automate these steps, reducing operator dependency and improving consistency across imaging systems [73,74]. Image segmentation is one of several important, time-consuming stages in digital workflows. Clinical expertise and significant time are required to delineate craniofacial structures manually. Recent advances in deep learning now allow CBCT datasets to be fully segmented automatically, including the mandible, maxilla, cranial bones, teeth, inferior alveolar nerve, maxillary sinus, and pathological lesions. Segmentation models using U-Nets, V-Nets, Mask R-CNNs, and transformers achieve high accuracy while substantially reducing segmentation time. Anatomical models derived from automated segmentation are used to create virtual surgical plans and implant designs. Using AI-based reconstruction, one can also restore native facial symmetry with greater precision than with traditional manual methods, such as mirror-image reconstruction [50,73].

Virtual surgical planning (VSP) has become a cornerstone of cranial reconstruction. Surgeons import anatomical models into dedicated planning software, where they can perform digital osteotomies, simulate tumor removal, and evaluate defects before entering the operating room. Surgeons can also use machine learning algorithms to estimate postoperative anatomical outcomes. These capabilities reduce planning variability and improve surgical precision [75,76]. After a craniofacial defect has been digitally defined, AI algorithms can reconstruct the missing anatomy. For bilateral defects and highly irregular defects, statistical shape modeling (SSM), atlas-based reconstruction, and deep generative networks are proving more effective than mirror-image reconstruction. Generative adversarial networks (GANs), variational autoencoders (VAEs), and diffusion models have shown considerable promise for generating realistic craniofacial geometries. In addition to improving aesthetics, AI-driven reconstruction techniques reduce subjective decision-making while restoring functionality [77,78]. Virtual reconstruction is followed by creating patient-specific implants (PSIs) using CAD software. Through automated implant contour generation, optimized implant thickness, and refined fixation screw positions, AI increasingly contributes to this stage. AI-assisted CAD platforms integrate anatomical constraints with biomechanical requirements, resulting in implants that closely match the patient’s native anatomy. Through generative design algorithms, thousands of possible implant geometries can be explored while simultaneously satisfying predefined objectives such as weight reduction, mechanical stability, surgical accessibility, and manufacturability. Therefore, AI-driven CAD improves surgical fit and implant precision while reducing design time [77,79].

Developing advanced craniofacial implants requires topology optimization. A topology optimization algorithm solves mechanical and biological constraints by determining the optimal distribution of material within an implant. Using AI, it can predict optimal porous architectures, minimize computational costs, and rapidly evaluate multiple design iterations. This approach achieves superior stress distribution, improved stiffness-to-weight ratios, controlled porosity, and enhanced vascularization potential in implants. Bioceramic scaffolds with interconnected porosity play an important role in osteogenesis and nutrient transport through these optimized architectures [80,81].

AI-driven digital workflows are incomplete without finite element analysis (FEA). CBCT data can be used to generate patient-specific finite element models that can simulate physiological loading conditions before implants are manufactured. AI algorithms further enhance FEA by predicting stress distributions, identifying failure regions, optimizing scaffold architecture, and estimating long-term implant performance. During implant development, machine learning surrogate models enable rapid iterative optimization by substantially reducing computational time and preserving prediction accuracy. By reducing design errors and increasing mechanical safety confidence before clinical application, such computational validation minimizes design errors [80,82]. After optimization, stereolithography files (STLs) are exported for implant manufacturing. Three-dimensional printing technologies can manufacture bioceramic implants with precise internal architectures. AI can detect manufacturing defects before fabrication using computer vision systems that monitor printing quality in real time. AI-assisted quality control improves manufacturing reproducibility and lowers production costs [80,83]. Digital twins are advancing personalized craniofacial reconstruction. Future digital twin platforms may enable prediction of biological processes such as bone remodeling, implant degradation, and osseointegration by integrating longitudinal clinical, imaging, and biomechanical data. However, reliable clinical prediction models are still under development and require extensive validation before routine application. In the future, digital twins could support personalized treatment planning and rehabilitation [71,84].

AI-driven workflows also include postoperative monitoring and outcome assessment. An AI algorithm analyzes postoperative CBCT images for implant positioning, bone regeneration, osseointegration, and potential complications. Personalized follow-up strategies can be recommended for patients at risk of implant failure using predictive analytics. An AI-powered clinical decision support system can help clinicians manage postoperative patients based on radiographic findings, patient-specific risk factors, surgical variables, and biomaterial characteristics. These intelligent systems are expected to improve long-term outcomes further as larger multicenter datasets become available and to facilitate continuous refinement of personalized treatment protocols [9,71].

**Figure 2:**
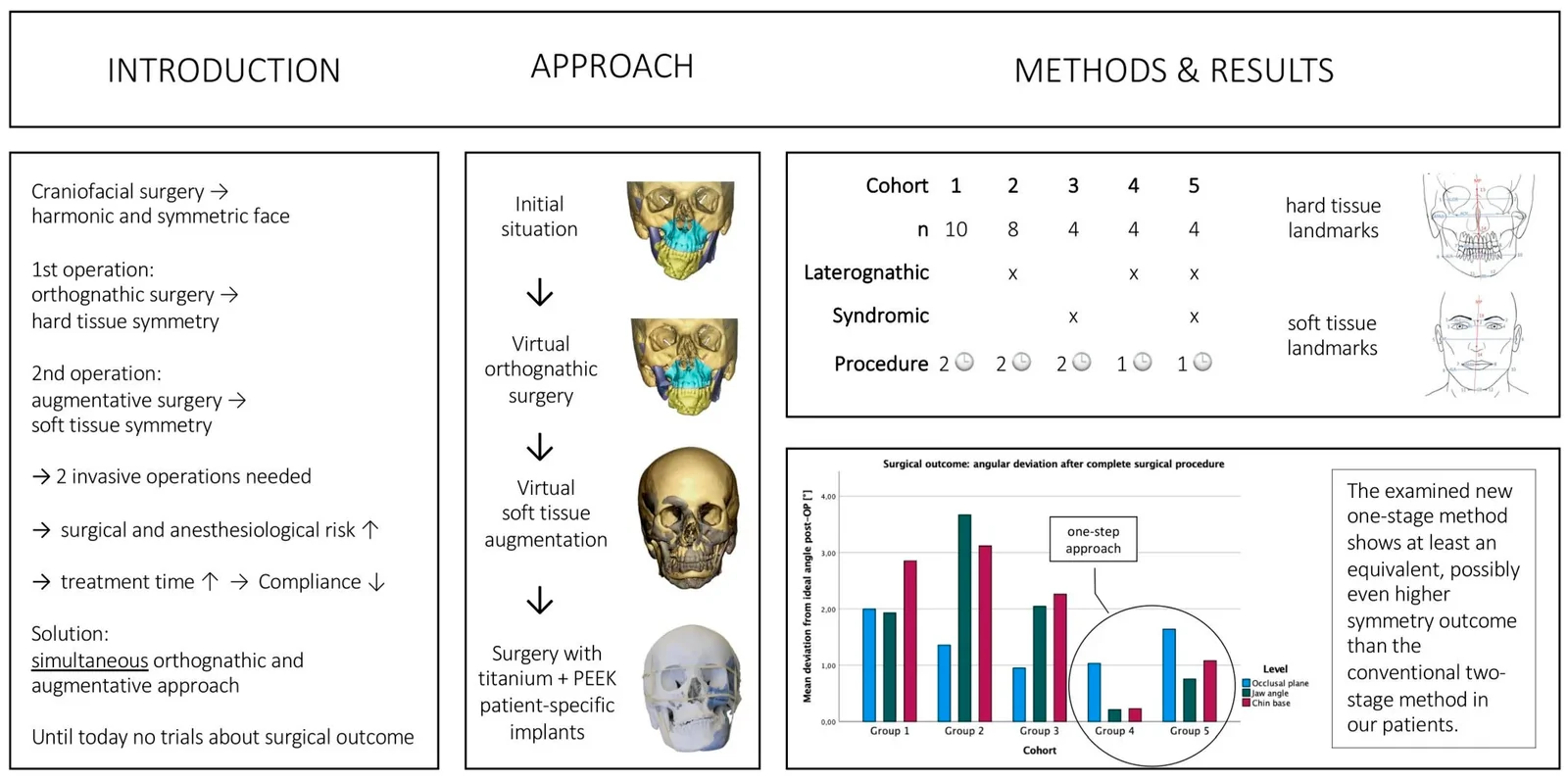
This graphic illustrates the study concept, digital workflow, and clinical outcomes associated with simultaneous PSI-based orthognathic surgery and PEEK bone augmentation. Using patient-specific titanium and PEEK implants, the virtual surgical planning process, the study cohorts, the evaluated landmarks, and postoperative symmetry results, this study indicates that the one-stage approach produces facial symmetry comparable to or better than that of the conventional two-stage approach [85].

In a study examining facial symmetry, Kerkfeld et al. (2022) [85] combined patient-specific implants (PSIs) with polyether ether ketone (PEEK) bone augmentation. They examined whether PSI-based orthognathic surgery with PEEK bone augmentation yielded better outcomes than conventional PSI-based orthognathic surgery. In addition, PEEK bone augmentation was classified as syndromic or non-syndromic. For each patient, a virtual surgery plan was developed using cone-beam CT (CBCT) to create cutting guides and osteosynthesis plates. In addition, PSI PEEK implants were created by superimposing deformed skulls over non-deformed skulls. Posteroanterior conventional radiographs and en-face photographs were obtained before and 9 months after surgery. A combination of orthognathic surgery and PEEK bone augmentation significantly improves facial symmetry. Following PSI-based orthognathic surgery, all patients’ bones aligned horizontally. PEEK bone augmentation improved soft- and hard-tissue underdevelopment resulting from syndromes (Figure 2).

Meto et al. (2025) [86] studied the evolution, applications, and future potential of CBCTs, AI, augmented reality (AR), and virtual reality (VR). They found that 3D imaging and digital imaging can reduce radiation exposure. AI applications enhance image interpretation, automate clinical tasks, and facilitate treatment simulations. AR and VR improve surgical training through engagement, competency-based learning, and real-time intraoperative guidance. Three-dimensional printing and portable imaging enhance accessibility and personalization in healthcare. By integrating CBCT, AI, AR, and VR, efficiency and precision can be improved, thereby improving dentistry and patient outcomes. Adopting this technology must be accompanied by research, standardization, and ethical practices to be effective [86].

## 7. Personalized Bioceramic Implants

In craniofacial bone tissue engineering, bioceramic implants combine patient-specific anatomical reconstruction with the intrinsic bioactivity of ceramic biomaterials. Unlike conventional off-the-shelf implants, personalized implants are designed directly from patient imaging data using CAD and fabricated with additive manufacturing. This digital workflow promotes biological bone regeneration rather than simply replacing missing tissue, matching complex craniofacial defects. Advances in AI have enabled automated image segmentation, implant geometry optimization, biomechanical prediction, and material selection, facilitating the development of next-generation patient-specific regenerative implants [87]. In terms of osteoconductivity, biocompatibility, and chemical composition, native bone closely resembles bioceramics. Especially suitable for craniofacial applications requiring rapid osseointegration and long-term biological stability, due to their ability to promote cell adhesion, proliferation, differentiation, and the mineralization of the extracellular matrix. Further advancements in scaffold engineering have enabled the fabrication of porous bioceramic structures that facilitate vascular infiltration and nutrient diffusion. AI-based algorithms and finite element analysis can optimize scaffolds for mechanical strength and biological performance, including pore size, porosity, surface roughness, and internal geometry [87,88]. The composition of calcium phosphate ceramics is similar to that of natural bone mineral, making them the most researched biomaterial for craniofacial bone regeneration. However, even though these materials are excellent osteoconductors, they do not trigger significant inflammatory responses when attached to bone [87,89]. Hydroxyapatite has advantages over other synthetic bioceramics, including biocompatibility, chemical stability, and high affinity for bone tissues. It has been demonstrated that 3D-printed HA scaffolds are particularly suitable for the reconstruction of non-load-bearing craniofacial defects thanks to their excellent osteoblast attachment and long-term dimensional stability. When defects are extensive, new bone may not fully replace HA because of its slow in vivo degradation. Researchers have therefore focused on tailoring scaffold porosity and incorporating AI-guided architectural optimization to enhance biological remodeling while maintaining structural integrity. Scaffold porosity is a critical parameter influencing biological performance. Interconnected pores facilitate cell migration, vascular infiltration, nutrient diffusion, and bone ingrowth; however, excessive porosity may compromise mechanical strength and structural stability. Therefore, optimal scaffold design requires balancing pore size, total porosity, interconnectivity, degradation rate, and mechanical requirements according to the anatomical location and functional demands of the defect [87,90,91].

In contrast to hydroxyapatite, tricalcium phosphate slowly degrades, allowing regenerative bone to replace it gradually. In addition to releasing calcium and phosphate ions during scaffold resorption, it also stimulates angiogenesis and osteogenesis. Although it has relatively low mechanical strength, it is not suitable for large load-bearing defects. Currently, pore architecture and scaffold geometry are optimized through computational modeling and AI-assisted design to maximize both mechanical stability and biological activity. The degradation behavior of bioceramics is strongly dependent on chemical composition, crystallinity, particle size, porosity, and local biological conditions. Hydroxyapatite demonstrates excellent stability and osteoconductivity but relatively slow resorption, whereas β-tricalcium phosphate exhibits faster degradation and replacement by newly formed bone. Biphasic calcium phosphate materials aim to combine these characteristics by adjusting the hydroxyapatite-to-β-TCP ratio to balance structural stability and biological remodeling [87,92]. Using biphasic calcium phosphate stabilizes structures and balances biodegradability. Based on clinical indications, researchers can customize degradation kinetics by adjusting the HA/β-TCP ratio. Recent studies have used AI to optimize phase composition and scaffold architecture for individualized craniofacial defects, thereby enhancing implant longevity and bone regeneration [87,93]. Ceramics based on calcium silicates, including wollastonite, akermanite, bredigite, and hardystonite, are attracting substantial attention for their superior bioactivity. The silicon ions released by these materials stimulate osteogenic differentiation, angiogenesis, and extracellular matrix mineralization. Moreover, calcium silicate ceramics integrate better with bone than other ceramics because they form apatite more rapidly after implantation. Recent studies also show that silicon-containing bioceramics promote macrophage polarization toward the pro-regenerative M2 phenotype through their immunomodulatory properties. To find optimal compositions that maximize both mechanical performance and biological function, AI-driven material optimization is increasingly being explored [87,94].

Among bioceramics, bioactive glass exhibits high surface bioactivity and can form hydroxycarbonate apatites after implantation. VEGF expression and osteoblast proliferation are accelerated through bioactive glass dissolution products. Using modern manufacturing techniques, it is possible to produce patient-specific porous bioactive glass scaffolds with highly interconnected architectures that facilitate vascularization throughout the implant. Recent studies have utilized AI-based computational models to optimize scaffold porosity and degradation profiles [87,95]. However, pure ceramics are intrinsically brittle, making them inapplicable in regions such as the craniofacial region that require high mechanical demands. To create composite bioceramics, researchers have combined ceramics with biodegradable polymers. In addition to being fracture-tough, these hybrid materials also exhibit excellent osteoconductivity. Composite scaffolds can also enhance regeneration by incorporating growth factors, antibiotics, stem cells, or extracellular matrix components. AI-assisted optimization can simultaneously evaluate multiple design parameters, such as ceramic/polymer ratio, pore geometry, and mechanical properties, to generate individualized scaffold configurations [65,87].

The development of personalized implants has become increasingly dependent on AI. Deep learning algorithms can generate automated craniofacial segmentation, 3D reconstruction of defect models, and patient-specific implant geometry in minutes. Generative design algorithms evaluate thousands of potential implant configurations while optimizing mechanical strength, stress distribution, weight reduction, and porosity. Machine learning models for scaffold degradation, bone integration potential, and implant reliability can also greatly reduce development time and improve implant reliability. With AI and finite element analysis, implants are virtually tested under physiological loading conditions before surgery, minimizing complications and enhancing long-term performance [87,96].

However, personalized bioceramic implants are still not widely adopted. Large scaffolds behave mechanically, have limited vascularization, are reproducible, may require sterilization, and require regulatory approval. AI models should be externally validated and trained on large, annotated datasets. Multimaterial 3D printing, smart bioceramics, digital twins, and closed-loop manufacturing systems guided by AI are all expected in the future. Such innovations will bring precision craniofacial tissue engineering closer to routine clinical practice, enabling implants to match patient anatomy and actively regulate bone regeneration and tissue remodeling [87,90,97].

It is important to distinguish the different product categories discussed throughout this review, as they serve different therapeutic purposes and follow distinct developmental and regulatory pathways. Permanent patient-specific implants, such as titanium and polyetheretherketone (PEEK) implants, are primarily designed to provide immediate structural reconstruction and long-term mechanical stability. These implants generally remain permanently in the patient and function as prosthetic replacements rather than regenerative materials. In contrast, bioceramic scaffolds are bioactive constructs intended to promote bone regeneration by providing an osteoconductive three-dimensional microenvironment that supports cellular infiltration, vascularization, and gradual tissue remodeling. Biodegradable graft substitutes, including calcium phosphate- and calcium silicate-based biomaterials, are designed to degrade progressively as they are replaced by newly formed bone, with degradation kinetics carefully matched to the healing process. Finally, cell-laden bioprinted constructs represent a distinct class of advanced tissue-engineered products that combine living cells with biomaterial-based bioinks to create biologically active constructs that promote tissue regeneration. Unlike acellular scaffolds or permanent implants, these products require preservation of cell viability during manufacturing, transportation, and implantation, resulting in substantially greater manufacturing complexity and more stringent regulatory oversight. Consequently, these four product categories should not be considered interchangeable, as they differ in biological function, intended clinical application, manufacturing workflow, sterilization strategy, regulatory classification, and current level of clinical translation [98,99,100].

## 8. Additive Manufacturing and Biofabrication

Additive manufacturing (AM), or 3D printing, has revolutionized craniofacial BTE by enabling fabrication of complex scaffolds and implants. From digital models, AM builds objects layer by layer, controlling scaffold geometry, pore size, porosity, interconnectivity, and internal architecture. A fully digital workflow for personalized craniofacial reconstruction includes AI, CBCT, CAD/CAM, and FEA. This approach allows patients to receive customized implants that match their specific anatomical defects and are designed to perform both mechanically and biologically. As a result of recent advancements in AM, personalized bone substitutes for maxillofacial and cranial defect repairs have become much more readily available for clinical use [3,101].

A variety of additive manufacturing technologies have been adopted for craniofacial bone regeneration, each offering unique advantages depending on the biomaterial, scaffold architecture, and clinical application. Among the most commonly used technologies for fabricating bone scaffolds are material extrusion, particularly fused deposition modeling (FDM) and direct ink writing (DIW). Polycaprolactone (PCL) can be extruded with FDM, whereas ceramic-rich pastes, hydrogels, and composite bioinks can be printed with DIW. With DIW, calcium phosphate- and calcium silicate-based bioceramic scaffolds can be fabricated while retaining bioactivity. AI algorithms are being used to optimize extrusion parameters, including nozzle diameter, extrusion pressure, deposition speed, and layer thickness, to improve scaffold fidelity and mechanical stability [102,103]. In addition to stereolithography, vat photopolymerization produces scaffolds with exceptional surface quality. This technique allows intricate craniofacial structures to be fabricated at the microscale by polymerizing a photosensitive resin layer by layer with light. Ceramic-filled photopolymerized resins enable the creation of highly detailed, patient-specific bioceramic constructs. AI-based optimization algorithms have been used to minimize printing defects and improve print quality [98,102]. By fusing powdered materials with lasers, powder bed fusion techniques form implants from powder. These technologies are well suited to titanium implants and porous metallic lattices. The combination of metallic frameworks with osteoconductive bioceramic coatings is increasingly being investigated to achieve mechanical strength and biological functionality. Combining computational optimization with AI-assisted topology design enhances implant performance and osseointegration [104,105]. Binder jetting enables the manufacture of high-porosity calcium phosphate and bioactive glass scaffolds without heating, using liquid binders. This technique facilitates bone ingrowth and vascularization by preserving interconnected porosity while allowing excellent geometric complexity. The latest developments in bioceramic implants have focused on optimizing binder compounds and sintering methods [92,106].

Engineered tissue constructs are made from living cells, biomaterials, bioactive molecules, and biological cues. Three-dimensional bioprinting can spatially distribute cells and biomaterials in predefined architectures, making it one of the most promising biofabrication technologies for craniofacial regeneration. Depending on the printing strategy, mesenchymal stem cells, osteoblasts, endothelial cells, growth factors, extracellular matrix components, and biodegradable hydrogels can be included. Using bioprinting and advanced biomaterials, vascularized bone constructs can support long-term tissue regeneration [107,108].

Commonly used bioinks include hydrogels made from gelatin methacrylate (GelMA), alginate, collagen, hyaluronic acid, and decellularized extracellular matrix. These hydrogels are extremely biocompatible and can encapsulate living cells. Although they are mechanically weak, they can often be reinforced with ceramic nanoparticles, calcium phosphates, bioactive glass, or synthetic polymers. For craniofacial bone regeneration, composite bioinks combine the biological advantages of hydrogels with structural stability [107,109].

AI is increasingly incorporated into additive manufacturing workflows to improve printing accuracy, efficiency, and reproducibility. To optimize printing speeds, extrusion pressures, nozzle temperatures, layer thicknesses, infill density, and scaffold orientations, machine learning algorithms analyze large datasets generated during printing. A predictive AI model can improve construction quality while reducing material waste. In addition, generative design algorithms create scaffolds with high porosities to facilitate cell infiltration and vascularization. Computational approaches reduce design iterations and accelerate personalized implant production [106,110].

Integrating AI into smart manufacturing systems now enables real-time quality control in biomedical additive manufacturing. The combination of computer vision systems and deep learning algorithms enables continuous monitoring of layer deposition, detection of printing anomalies, and identification of dimensional inaccuracies during fabrication. Digital twins and closed-loop feedback systems ensure scaffold quality is consistently maintained by automatically adjusting printing parameters when errors are detected. Improved process standardization and traceability will facilitate the regulatory approval of patient-specific implants using these intelligent manufacturing platforms [106,111].

Although considerable progress has been made, several technical challenges remain before additive manufacturing and biofabrication can be widely adopted in craniofacial bone tissue engineering. In particular, for large load-bearing defects, it remains difficult to achieve an optimal balance among scaffold porosity, mechanical strength, degradation kinetics, and biological performance. The large engineered constructs must also be vascularized, printed biomaterials must be adequately standardized, manufacturing processes must be scalable, and regulatory requirements must be met. AI-guided autonomous manufacturing; multimaterial printing; stimuli-responsive biomaterial printing in four dimensions (4D); and fully integrated digital workflows combining CBCT imaging, generative AI, computational biomechanics, robotic manufacturing, and personalized regenerative medicine are expected to be among the next few developments. As a result, additive manufacturing will become a cornerstone technology for craniofacial reconstruction in the near future [3,112].

## 9. Clinical Translation and Applications

Clinical translation of craniofacial BTE from laboratory research to patient-specific therapies has been accelerated by integrating AI, CBCT, CAD/CAM, FEA, and additive manufacturing. CBCT images can be converted into anatomically accurate 3D models, allowing surgeons and engineers to create precise surgical plans. With AI-assisted reconstruction, operative time is reduced, surgical accuracy is enhanced, functional and esthetic outcomes are improved, and complications caused by intraoperative implant modifications are minimized [3,8,21].

In trauma, osteoradionecrosis, congenital deformities, or oncologic resection, mandibular defects present significant reconstructive challenges because the mandible plays key roles in chewing, speaking, and maintaining the airway. Large defects remain best treated with vascularized fibular free flaps, but prolonged surgical procedures and donor-site morbidity have prompted the development of patient-specific alternatives. With AI-assisted digital workflows, mandibular anatomy is segmented from CBCT, automated defects are reconstructed, mirror images are generated, and virtual surgery is planned. FEA can predict stress distribution, optimize implant geometry, and identify anatomical landmarks using machine learning algorithms. Compared with conventional stock implants, titanium fixation systems and porous 3D-printed bioceramic scaffolds exhibit superior adaptation. In clinical studies, AI-assisted CAD/CAM workflows have been shown to improve occlusal restorations, reduce intraoperative adjustments, reduce operative time, and improve surgical precision with patient-specific implants [3,83,113].

Moreover, calcium phosphate and calcium silicate-based scaffolds have shown promising osteoconductive properties, especially when combined with osteogenic cells or growth factors. AI-assisted scaffold optimization can achieve maximal vascular infiltration while retaining sufficient mechanical stability to support load-bearing [8,114].

A maxillary defect requires more complicated reconstruction than a mandibular defect since the maxilla supports the orbit, masticates, speaks, and supports the nasal cavities. After tumor resection, extensive defects often require individualized reconstruction to restore skeletal integrity and soft tissue support. With CBCT-based digital planning, surgeons can accurately visualize maxillary anatomy and segment defects with AI assistance. Surgeons can simulate osteotomies before surgery and determine implant placement and facial contours postoperatively with virtual surgical planning. Custom bioceramic implants can accurately reproduce the complex anatomy of the maxilla through CAD/CAM and additive manufacturing. Recent advances in deep learning have enabled automated reconstruction of complex maxillary defects using generative design algorithms that incorporate anatomical symmetry, biomechanical requirements, and prosthetic requirements. Compared with conventional reconstruction techniques, these technologies enable highly individualized implant designs and have demonstrated encouraging esthetic and functional outcomes in selected clinical reports; however, comparative studies with long-term follow-up are still required [5,8,15,107].

Implant manufacturing remains a successful clinical application in cranioplasty. Because the calvarium’s geometry is relatively predictable, digital reconstruction is an ideal technique for reconstructing defects caused by trauma, decompressive craniectomy, congenital malformations, or tumor resections. AI can automatically segment CBCT or CT datasets, identify defects, mirror images, and generate implants. Several AI-based algorithms have been developed to reduce manual modeling time while improving anatomical accuracy. Patient-specific reconstruction encompasses several distinct product categories. Permanent implants, including titanium and PEEK devices, are primarily intended for structural replacement, whereas patient-specific bioceramic scaffolds function as regenerative templates for new bone formation. Biodegradable graft substitutes provide temporary structural support while gradually resorbing during healing, whereas cell-laden bioprinted constructs are living tissue-engineered products designed to promote tissue regeneration actively. Although all may be generated through digital workflows, they differ fundamentally in biological purpose, manufacturing requirements, and regulatory approval pathways [8,115,116].

Asymmetric facial features, such as diplopia, enophthalmos, and impaired ocular motility, can result from even minor errors in orbital fracture implant design. Using deep learning algorithms, CBCT datasets can be automatically segmented and fracture boundaries identified. By reconstructing the mirror image of the unaffected orbit, it is possible to design personalized implants for each patient. AI-based finite element simulation can further optimize implant thickness and performance while minimizing implant weight. A 3D-printed bioceramic and titanium implant offers excellent anatomical conformity, shorter operative time, and more accurate orbital volume restoration than conventional prebent mesh systems. These improvements lead to better vision and better esthetic recovery [5,117].

Ankylosis, degenerative diseases, trauma, and tumor resection frequently require temporomandibular joint replacement. Because of the TMJ’s complex biomechanics, computer modeling is essential for reconstruction. Automated segmentation and CBCT imaging allow accurate reconstruction of the mandibular condyle and glenoid fossa. AI-assisted CAD systems can tailor the TMJ prosthesis to a patient’s anatomy, and finite-element analysis can predict stress distributions. Personalized TMJ prostheses improve mandibular function, reduce postoperative complications, and enhance long-term stability. By analyzing each patient’s biomechanical loading conditions, AI-driven predictive models can extend implant longevity [8,28].

Dental implants are planned using CBCT imaging, guided surgery, and bone analysis. AI algorithms can automatically assess alveolar bone quality and identify anatomical risk structures for optimal implant placement. AI-assisted workflows can design CAD-assisted bioceramic scaffolds to augment ridges in cases of severe alveolar bone loss. In addition to promoting vascularization and osseointegration, 3D-printed calcium phosphate scaffolds are mechanically robust. Machine learning models are increasingly being applied to predict implant stability, osseointegration, peri-implant bone remodeling, and long-term implant survival. With these predictive capabilities, implant rehabilitation may be more successful over the long term and support personalized treatment planning [5,8,118].

Although AI-assisted digital workflows have shown promising results in computational studies, preclinical models, phantom investigations, and early clinical applications, their translation into routine craniofacial reconstruction remains in its early stages. Existing evidence consistently indicates that implants designed with AI-integrated workflows are more anatomically accurate, achieve greater surgical precision, take less time to perform, and are more patient-friendly than conventional reconstructions. Future clinical implementation will require multicenter prospective clinical trials, standard AI validation protocols, regulatory approval of adaptive algorithms, and interoperability among imaging and manufacturing platforms. As digital twins, robotic surgery, real-time intraoperative navigation, and AI-driven predictive analytics are integrated into routine clinical practice, personalized craniofacial reconstruction will further advance [3,5,8,115,119].

## 10. Challenges and Limitations

The widespread adoption of craniofacial bone tissue engineering remains limited by difficulties in integrating AI into digital workflows, including technical, clinical, regulatory, and ethical challenges. To translate AI-assisted, personalized bioceramic implants into routine clinical practice, these limitations must be addressed. Developing AI models is hindered by the scarcity and poor quality of available imaging datasets. Deep learning algorithms typically struggle to achieve robust performance without large, diverse, and accurate datasets. Craniofacial CBCT datasets often come from a single institution, are acquired using different imaging protocols, and are manually annotated by experts, resulting in limited sample diversity and significant variability between observers. As a result, AI models cannot be generalized across patients, imaging devices, and clinical settings. A further risk of algorithmic bias arises from the underrepresentation of rare craniofacial deformities and complex reconstructive cases in existing datasets. Moreover, AI algorithms must be interpretable and accurate. Deep neural networks excel at image segmentation, landmark detection, and implant design, but many of their predictions remain opaque. Regulatory approval is complicated by a lack of transparency, which limits clinician confidence. Explainable AI (XAI) is still too early to integrate into clinical workflows. CBCT image quality varies widely. Metallic restorations, patient motion, beam hardening, and scanner calibration differences can also affect downstream processes, such as virtual surgical planning. Reproducible and consistent AI performance requires robust preprocessing algorithms and standard imaging protocols. Customized bioceramic implants present additional manufacturing challenges. Although additive manufacturing can produce highly customized scaffolds with complex geometries, maintaining dimensional accuracy, homogeneous porosity, adequate mechanical strength, and reproducible material properties remains a technical challenge. Standardizing manufacturing protocols and quality assurance systems is important to minimize the impact of variability in printing parameters, sintering conditions, and material composition on scaffold performance. Biological limitations also warrant consideration. The osteoconductive and biocompatibility properties of bioceramics are often undermined by their low fracture toughness, brittle mechanical properties, and limited intrinsic osteoinductive capacities. In large load-bearing craniofacial defects, balancing scaffold degradation, mechanical stability, vascularization, and new bone formation remains challenging. In addition, most AI-assisted implant optimization studies rely on preclinical animal models or computational simulations rather than long-term clinical evidence. SaMDs are increasingly used for diagnosis, treatment planning, and implant design, requiring rigorous validation, cybersecurity measures, and ongoing performance monitoring. AI-assisted clinical decisions, data ownership, and intellectual property remain ethically and legally unresolved. Data privacy and cybersecurity are not the only obstacles to widespread implementation. Large-scale datasets containing sensitive patient information are commonly required for AI models. Data privacy regulations, secure data sharing, anonymized data, and federated learning strategies will be crucial to maintaining patient confidentiality. Economics should also not be overlooked. Establishing an integrated digital workflow requires CBCT systems, high-performance computing infrastructure, specialized software, 3D printers, and multidisciplinary expertise. These costs may be unaffordable for low- and middle-income healthcare settings, and cost-effectiveness analyses are lacking. AI-driven craniofacial reconstruction requires collaboration between surgeons, radiologists, biomedical engineers, materials scientists, and computer scientists. Technical differences, interoperability limitations, and the lack of standardized clinical workflows hinder efficient, broader multidisciplinary collaboration. To be successfully translated into routine patient care, AI-powered digital workflows for craniofacial bone tissue engineering must overcome challenges in data quality, algorithm transparency, manufacturing reproducibility, biological performance, regulatory compliance, cybersecurity, and clinical validation. Future research should emphasize multicenter prospective studies, standardized benchmarking protocols, explainable AI methodologies, and interoperable digital platforms for AI-enabled personalized regenerative medicine.

Although recent advances in AI have demonstrated impressive technical performance, many published models remain insufficiently validated for routine clinical implementation. Most algorithms have been developed and tested using retrospective datasets from single institutions, limiting their external validity when applied to different patient populations, imaging protocols, CBCT devices, and clinical environments. Dataset heterogeneity and inconsistent annotation standards may substantially affect model performance. At the same time, underrepresentation of uncommon craniofacial deformities, pediatric cases, and diverse ethnic populations may introduce algorithmic bias and reduce fairness across patient groups. Consequently, external multicenter validation using standardized benchmark datasets should become a prerequisite before widespread clinical adoption.

Equally important, current AI systems should be regarded as decision-support tools rather than autonomous clinical decision-makers. Human supervision remains essential for confirming image segmentation, reviewing virtual surgical plans, verifying implant designs, and interpreting AI-generated recommendations, particularly in anatomically complex cases or when image quality is compromised. From a manufacturing perspective, the reproducibility of patient-specific bioceramic implants remains a major translational challenge because printing parameters, raw material characteristics, post-processing procedures, and sterilization protocols can all influence scaffold geometry, mechanical properties, and biological performance. Standardized manufacturing workflows, rigorous quality-control procedures, and compliance with Good Manufacturing Practice (GMP) requirements are therefore essential to ensure consistent implant quality.

Furthermore, successful clinical translation will require compliance with evolving regulatory frameworks governing AI-enabled Software as a Medical Device (SaMD), patient-specific implants, and additive manufacturing technologies. Regulatory agencies increasingly require evidence of algorithm transparency, continuous post-market monitoring, cybersecurity, risk management, and clinical validation before approval. Despite encouraging findings from computational studies, preclinical investigations, and small pilot clinical studies, robust evidence from large multicenter randomized or prospective clinical trials remains scarce. Therefore, the long-term safety, effectiveness, cost-effectiveness, and clinical superiority of AI-assisted personalized bioceramic reconstruction over conventional treatment approaches have not been conclusively established.

## 11. Future Perspectives

Despite rapid technological progress, most AI-enabled craniofacial reconstruction approaches remain in the developmental or early translational phase. Current evidence primarily supports their feasibility and potential advantages rather than definitive superiority over conventional approaches. Future research should focus on prospective multicenter trials, standardized evaluation frameworks, and long-term clinical monitoring to establish their true therapeutic value.

AI, advanced medical imaging, computational modeling, and additive manufacturing are enabling a new era of precision and personalized regenerative medicine. In the next decade, several technological innovations are expected to further revolutionize digital workflows, including AI-assisted image analysis, virtual surgical planning, and patient-specific implant fabrication. Digital twins are dynamic virtual representations of individual patients that incorporate imaging, clinical, biomechanical, and biological information. Simulating tissue remodeling, implant performance, and bone regeneration with digital twins is more accurate than with conventional 3D anatomical models. CBCT-derived anatomical information and biomechanical simulations could help surgeons predict postoperative outcomes, optimize implant geometry, and tailor treatment strategies. AI and digital twin technology will enable real-time surgical planning and longitudinal monitoring of tissue regeneration, ultimately improving clinical outcomes. The industry is also set to be transformed by foundation models. Machine learning will be able to design multiple implants that meet anatomical, biomechanical, and biological constraints without manual optimization or engineer-defined parameters. Using generative design algorithms, thousands of scaffold architectures can be rapidly explored, with porosity, mechanical strength, nutrient diffusion, degradation profiles, and mechanical properties optimized simultaneously. With the development of large multimodal foundation models, it may be possible to integrate radiographic images, surgical records, biomaterial databases, and patient outcomes into unified decision-support systems that assist clinicians throughout the entire reconstruction process. Advances in machine learning-guided biomaterial discovery are expected to accelerate the development of next-generation bioceramics. Research costs and development time are significantly reduced when AI algorithms analyze extensive experimental datasets to predict the biological performance of novel material compositions before laboratory synthesis. Machine learning models are increasingly used to optimize calcium phosphate ceramics, bioactive glasses, calcium silicate-based materials, and composite scaffolds by predicting mechanical properties, degradation kinetics, ion release profiles, osteogenic potential, and antibacterial properties. AI -driven material discovery may lead to intelligent biomaterials tailored to the needs of individual patients and defects.

As 4D printing advances, printed constructs will also change their shape, mechanical properties, or biological function in response to external stimuli such as temperature, pH, moisture, magnetic fields, or biochemical signals. The regenerative microenvironment can be dynamically adapted with 4D-printed bioceramic scaffolds, improving vascularization, mechanical stability, and tissue maturation. AI algorithms will be crucial to designing programmable scaffolds and predicting stimulus-responsive behavior during the healing process. AI is also advancing in bioprinting and biofabrication. Future craniofacial implants may incorporate living cells, extracellular matrix components, growth factors, and vascular networks. Aside from optimizing bioink compositions, printing parameters, and cell distributions on scaffolds, AI can also predict cell viability. Integrating AI-assisted bioprinting and personalized regenerative therapies may improve bone regeneration while reducing complications associated with conventional implants. Digital workflows can also benefit from robotics and autonomous surgical systems. AI-assisted robotic surgery can place highly accurate implants, automatically guide osteotomies, and perform intraoperative navigation using continuously updated CBCT images. Using augmented, virtual, and mixed reality, surgeons can now perform procedures with unprecedented precision. This approach can reduce surgical errors, shorten operative time, and improve reconstructive accuracy, especially for complex craniofacial defects. Another area that needs development is continuous learning healthcare systems. Using federated learning instead of static AI models that learn only from limited datasets, future algorithms will continually improve, allowing institutions to train models collaboratively without revealing sensitive patient information. Privacy-preserving approaches will enable the development of robust, generalizable AI systems across imaging devices, healthcare facilities, and patient populations. Multicenter databases will be standardized, imaging formats will be interoperable, and international reporting guidelines will be developed to facilitate this transition. AI-driven digital workflows will face several challenges before becoming routine clinical practice. Developing reliable deep learning models requires large, high-quality, annotated datasets. Barriers remain, such as algorithm transparency, explainability, cybersecurity, regulatory approval, ethical governance, and medico-legal accountability. AI systems should provide clinicians with a deeper understanding of why automated recommendations are made, increasing their trust in them. A standardized validation protocol is also needed to ensure the safety, reproducibility, and effectiveness of AI-enabled medical devices. In the future of craniofacial BTE, AI, multimodal imaging, computational biomechanics, intelligent biomaterials, additive manufacturing, and precision surgery will be integrated. An end-to-end workflow could transform reconstruction into a proactive, preventative, and highly personalized therapeutic approach. In the future, patient-specific craniofacial regenerative therapies will be possible through collaboration among clinical researchers, biomaterials scientists, engineers, computer scientists, and regulatory authorities.

## 12. Conclusions

AI represents a promising enabling technology for craniofacial bone tissue engineering by integrating CBCT-based imaging, computational modeling, CAD/CAM, and personalized bioceramic manufacturing. Current evidence suggests that AI-assisted workflows may improve planning accuracy, design optimization, and procedural efficiency; however, definitive evidence regarding long-term clinical outcomes remains limited. Future progress will depend not only on technological innovation but also on rigorous external validation of AI models, standardized multicenter datasets, reduction in algorithmic bias, and continued expert clinical oversight throughout the digital workflow. Equally important are reproducible manufacturing processes, internationally harmonized regulatory pathways, and high-quality prospective clinical trials that can demonstrate long-term safety, effectiveness, and cost-effectiveness. Addressing these challenges will be essential for translating AI-assisted craniofacial reconstruction from promising research into routine evidence-based clinical practice.

## Data Availability

No new data were created or analyzed in this study. Data sharing is not applicable to this article.
